# Multimorbidity: Addressing the Elephant in the Clinic Room

**DOI:** 10.3390/healthcare13101202

**Published:** 2025-05-21

**Authors:** David Cosio

**Affiliations:** Jesse Brown VA Medical Center, Chicago, IL 60612, USA; david.cosio2@va.gov; Tel.: +1-312-569-8703

**Keywords:** multimorbidity, health psychology, chronic pain

## Abstract

Multimorbidity is the conjoint presence of multiple conditions in patients, which is a public health problem. Multimorbidity is like the elephant in the clinic room because it remains the unaddressed challenge we face in healthcare. Clinical health psychology has a role to play in this undertaking because it recognizes the intersection and interface of concurrent mental and/or behavioral problems and physical diseases. The current article will define multimorbidity, describe current statistics, how it differs from comorbidity, how to use the biopsychosocial model, and ways in which clinical health psychologists can manage and prevent it in their clinics. A model of how to address multimorbidity will be shared using the role of a clinical health psychologist working in a multidisciplinary pain clinic in a hospital setting serving patients who are socioeconomically disadvantaged.

## 1. Introduction

Multimorbidity is the situation in which the presence of several chronic health conditions (i.e., physical and mental disorders) are all treated to affect the patient’s global health status [1]. Ongoing persistent conditions, such as chronic pain and substance misuse, are also included [2]. This phenomenon is an increasing public health problem. The goal of the current manuscript is for current and future clinical health psychologists to learn about multimorbidity and how they can make important contributions in addressing this public health problem. The article will describe current statistics related to multimorbidity, the difference between multimorbidity and comorbidity, how to use the biopsychosocial model to address multimorbidity, and ways in which current and future clinical health psychologists can manage and prevent multimorbidity in their clinics. A model for addressing multimorbidity will be discussed to guide future quality improvement initiatives

### 1.1. Current Statistics

The prevalence of patients presenting with multiple health conditions has markedly increased in the last few decades. It has been estimated that one in four Americans has two or more health conditions [3]. Further, people who come from a low SES tend to experience multimorbidity with inequity, more disability, declines in functionality, worsened quality of life, and greater health care costs [4]. This problem is not only restricted to the socioeconomically deprived; it also negatively affects women, people of color, and the elderly [4]. However, the problem is not restricted only to these populations. For example, research has shown that by the age of 30, 20–30% of patients live with multiple medical conditions [5]. There is also evidence of disproportionate multimorbidity among Veterans who are middle aged [6].

### 1.2. Multimorbidity Versus Comorbidity

It is important to clarify the distinction between the terms multimorbidity and comorbidity. Comorbidity refers to the grouping of additional diseases beyond a primary condition that a single provider is addressing [7]. For example, an oncologist who is treating a patient for cancer may acknowledge other health conditions, but they are not the primary interest nor are addressed by the same physician. They may consider, however, how they may affect the prognosis of cancer [8]. The current healthcare system operates in silos, or single-condition foci. There is a need to move beyond this strategy. Multimorbidity is like the elephant in the clinic room because it remains the unaddressed challenge we face in healthcare [9]. Multimorbidity is the conjoint presence of multiple chronic physical and mental conditions that are treated by the same provider with the intention to affect a patient’s global health status [1]. Multimorbidity occurs with multifactorial causes or risk factors, including shared genes [10], traditional medical risk factors (e.g., hypertension) [11], certain psychological disorders [12], and behavioral risk factors (e.g., tobacco use) [13]. Recent systematic reviews highlight that individuals with multimorbidity experience higher mortality and hospitalization rates, longer hospital stays, poorer quality of life, functional limitations, and increased psychological stress [14]. The current challenges in managing multimorbidity include fragmented care, polypharmacy, and a lack of research on the specific needs of this population [15]. Clinical health psychology has a role to play in this endeavor because it identifies the overlap and interaction of comorbid mental/behavioral problems and physical diseases [10]. For example, a clinical health psychologist who is treating a patient for chronic pain in a primary care setting may also educate and treat that patient’s depression, anxiety, PTSD, and substance use disorder and/or lifestyle habits (tobacco use, weight loss, sleep disorder, etc.) to improve their global health status.

### 1.3. The Biopsychosocial Model

Several different theoretical and alternative models can be found in the literature that address multimorbidity. These include the Lifecourse Model of Multimorbidity Resilience [16], the Integrated Model of Multimorbidity and Symptom Science [17], and Network Medicine [18], to name a few. Amongst these models is the biopsychosocial model. All these models represent a move away from a fragmented, disease-specific approach towards a more comprehensive and person-centered approach that recognizes the complexity of multimorbidity. A biopsychosocial approach considers the whole person as the object of assessment and treatment and rewrites the rules and expectations about treatment. The biopsychosocial model recognizes that patients rarely experience only one chronic health condition, and they exists in an interactive, multilevel biological, psychological, and social environment, which includes culture [19]. It suggests that the provider not only identify biomedical features but also encourages them to focus on the psychological and social factors of the patient’s life. These factors are believed to be responsible for the persistence of the disease [20]. Biopsychosocial methods acknowledge the need to treat the entire person, mind and body, tend to apply comprehensive psychosocial diagnostic strategies, are long-term in duration, assess the effects of the illness, and define disease as a complex problem. People who suffer from chronic diseases are also persuaded to take a more active role in their treatment. It is recommended patients move from a passive “disempowering” attitude to a more active “empowering” stance. This is different from the biomedical approach, which is used during the acute phase of illness. The traditional, biomedical model used to treat acute disease has proven to be unacceptable in the treatment of chronic disease. Biomedical methods separate the body and mind relationship, tend to employ technology as its diagnostic strategy, are short-term in duration, assess the cause, and define disease as a symptom. Clinical health psychology is equipped to address these issues because it is based on the biopsychosocial model.

### 1.4. The Role of Clinical Health Psychologists

Clinical health psychology has been forward thinking in its identification of the relationship between psychological and physical health [19]. Clinical health psychologists can contribute basic and applied knowledge relevant to several aspects of care that are more complicated with multimorbidity, including patient–provider communication, shared medical decision-making, and patient compliance/adherence. They function as an integral part of the medical team but can also play a liaison role. They can also capitalize on their experience with evidence-based interventions (e.g., biofeedback, relaxation training, stress inoculation, and cognitive behavioral strategies). They are able to implement behavioral interventions at multiple levels of influence, including the patient, the caregiver, and the healthcare provider [21]. Clinical health psychologists are trained in the ability to control one’s thoughts, emotions, impulses, and behaviors, which is an integral element at the primary (i.e., primary care), secondary (i.e., specialty consultation), and tertiary (i.e., advanced medicine) levels of care [19]. They also possess the unique education to treat both mental and/or behavioral disorders and the self-management of chronic illness, such as chronic pain.

## 2. Pain Management Clinic as a Model

The role of a clinical health psychologist working in a multidisciplinary pain clinic in a Department of Veterans Affairs (VA) hospital setting serving patients who are socioeconomically disadvantaged in one year will be shared as a model of how to address multimorbidity. Such a model has the potential to bring greater accountability to the delivery of mental health care and to inform quality improvement efforts.

Every patient who comes to the pain clinic and/or who is directly referred to the pain psychology clinic is offered an hour-long initial assessment with the clinical health psychologist. Patients who are referred may include those who have failed medical/surgical treatment; exhibited an overreliance on medications/therapies; displayed pronounced inactivity; suffered from significant depression or anxiety related to his/her pain; demonstrated inadequate coping skills; and/or appeared receptive to adopting a self-management approach to pain management.

Veterans at the sample institution have mixed idiopathic chronic, noncancer pain conditions, including back, neck, extremity, head, and fibromyalgia/soft tissue pain. Caucasian and Hispanic/Latinx patients are represented, but most are African American. There is also a large number of female Veterans, but most are males. Veterans tend to be between the ages of 55 and 64 years old, or are generally considered to be in the “middle age” demographic [22].

## 3. Results

**Figure 1:**
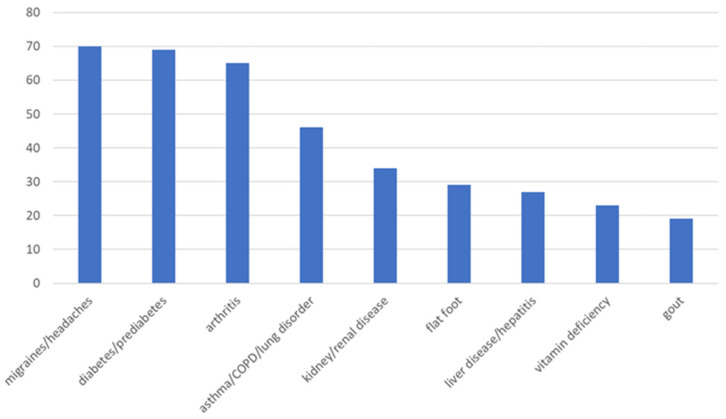
Frequencies of comorbid medical conditions present in a pain clinic sample.

Approximately 50% of patients who come to the pain clinic report more than three co-occurring medical conditions, and less than 10% do not report another medical condition. The three most prevalent co-occurring medical conditions in the one year sample from the pain clinic include migraines, type-2 diabetes, and arthritis (all about 30% each) (see Figure 1).

**Figure 2:**
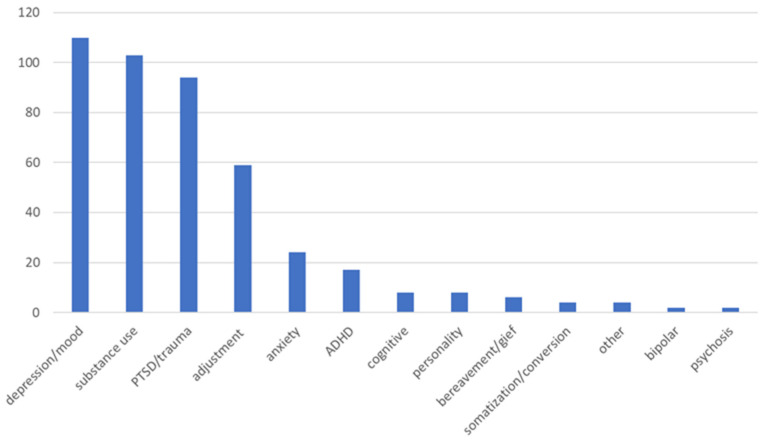
Frequencies of comorbid mental health conditions in a pain clinic sample.

Approximately 20% of patients who come to the pain clinic report more than three co-occurring mental health conditions, and less than 10% did not report a mental health condition. The three most prevalent co-occurring mental health conditions in the one year sample from the pain clinic include depression, substance use, and PTSD (all about 40% each) (see Figure 2).

**Figure 3:**
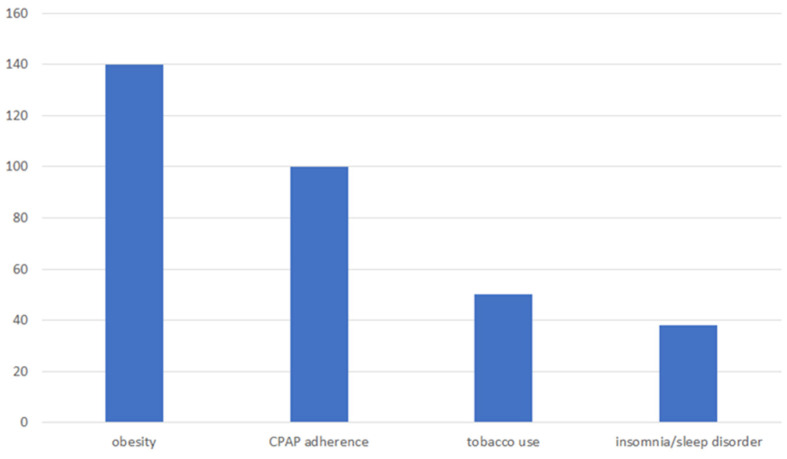
Frequencies of comorbid unhealthy lifestyle habits in a pain clinic sample.

Patients who come to the pain clinic report needing assistance with lifestyle habits (obesity, sleep apnea, tobacco, and insomnia or other sleep disorders) (see Figure 3).

Over 50% of patients coming to the pain clinic were obese, with a BMI > 30. About 10% of those patients were reportedly already engaged in weight management services when approached about addressing this concern. Approximately 50% of those patients were educated about the negative effects of weight on pain and declined further services versus almost 40% of patients who agreed to further weight loss intervention. Over 40% of patients coming to the pain clinic were nonadherent to their CPAP machine to address sleep apnea (OSA). About 15% of those patients were reportedly already engaged with the sleep clinic (e.g., new masks, sleep study, need a CPAP machine, etc.) when approached about addressing this concern. Approximately 60% of those patients were educated about the negative effects of CPAP noncompliance on pain and declined further services versus almost 25% of patients who agreed to further CPAP adherence intervention. Over 20% of patients coming to the pain clinic reported using tobacco (smoke, vape, or chew). Approximately 80% of those patients were educated about the negative effects of tobacco use on pain and declined further services at that time versus almost 20% of patients who agreed to further smoking cessation intervention. An additional 15% of patients coming to the pain clinic reported insomnia or other sleep disorders. Approximately 60% of those patients were educated about the negative effects of disturbed sleep on pain and declined further services at that time versus almost 40% of patients who agreed to further sleep intervention.

About 75% of the patients in the current pain clinic sample agreed to a referral to a pain education program. Approximately 40% of those patients participated in the education program. The average patient attended almost half of the 12-week program. Approximately 65% of patients in the current pain clinic sample were already engaged in mental health services, while 10% were identified as not having any mental health concerns. Almost 25% of patients in the current pain clinic sample were identified as needing further mental health services at that time. Only about 40% of those patients agreed to a referral for long-term, individual psychotherapy or a psychiatric consultation. About 70% of patients in the current pain clinic sample were identified as having a centralized sensitization syndrome and received education about the positive effects of neurostimulation. Only about 50% of those patients agreed to further treatment with neurostimulation. Over 50% of patients in the current pain clinic sample agreed to a referral to a pain group after receiving education about it. Close to 60% of those patients participated in a pain group. The average patient attended about four sessions of a pain management group. Approximately 8% of patients in the current pain clinic sample were identified as having an untreated substance use disorder. Only about 35% of those patients agreed to a referral to a specialized addiction service after receiving education about it.

## 4. Discussion

The current findings indicate the three most prevalent co-occurring medical conditions in the current sample from the pain clinic include migraines, type-2 diabetes, and arthritis. This is similar to what is reported in the literature. According to past literature, the most prevalent clusters of co-occurring conditions include cardiometabolic (e.g., type-2 diabetes), neuropsychiatric (e.g., migraines), and musculoskeletal (e.g., arthritis) [23]. The current findings also indicate the three most prevalent co-occurring mental health conditions include depression, substance use, and PTSD. Past research findings indicate that pain symptoms are associated with at least a double increased risk for comorbid depression [24]. Up to about 50% of patients who suffer from chronic pain also report a comorbid substance use disorder [25]. Past research findings suggest that Veterans referred to a pain clinic (about 10%) and patients receiving care at a multidisciplinary pain center (10%) meet the criteria for PTSD [26]. These comorbid conditions can be addressed in a pain clinic by using the following three-pronged approach: (1) providing mental health treatment, (2) facilitating lifestyle habit modifications; and (3) providing pain education programming.

*Mental Health Treatment.* A strong relationship between chronic pain and mental health disorders has been found in numerous studies [27]. There are several options for treatment of mental health disorders available, including individual psychotherapy, group therapy, self-help modalities, psychiatric medications, and neurostimulation. Past research investigating CES neurostimulation has found a possible beneficial effect of modest size in patients with fibromyalgia-like symptoms and mood disorders [28].

*Group Psychotherapy*. Acceptance and Commitment Therapy (ACT) and traditional Cognitive–Behavioral Therapy (CBT) are among the most applied behavioral medicine interventions for chronic pain. Past research has found that ACT is as effective as traditional CBT in the treatment of chronic pain among different populations, including Veterans [29]. However, ACT and CBT are not effective for all patients and may not have comparable effects [29,30,31]. There are theoretical differences between ACT and traditional CBT, including the role of cognitions and emotional regulation strategies in therapy, but they tend to overlap behavioral techniques and strategies.

*Specialized Addiction Services*. Patients who suffer from a substance use disorder (SUD) have been shown to be at greater risk for aberrant medication-related behaviors, which can negatively affect pain management. Patients with comorbid SUDs are also potentially more difficult to treat and add additional concerns about other comorbidities (depression, anxiety, etc.). Combination use of medication and psychotherapy are important elements of an overall treatment program, which may include detoxification, treatment, and relapse prevention [32]. There is evidence that suggests that psychotherapy enhances the efficacy of medications and facilitates patients staying longer in treatment. Addiction treatment can be delivered utilizing different behavioral approaches in different outpatient settings. For example, some highly structured residential treatment programs require patients to stay at a residence anywhere between half a year to a year.

*Lifestyle Habit Modification*. The current findings indicate patients who come to the pain clinic report needing assistance with lifestyle habits (obesity, sleep apnea, tobacco, and insomnia or other sleep disorders). According to the CDC, about 2/3 of US adults are overweight or obese and are at increased risk for musculoskeletal disease [33]. Veterans not using the VA and nonveterans have lower rates of obesity than Veterans who use the VA for health care. About 43% of the general population are of normal weight compared to only 28% of Veterans who receive health care at the VA [34]. Obstructive sleep apnea (OSA) is very common among middle-aged adults, affecting anywhere from 2% to 15% [35]. Past research has shown that Veterans may be at increased risk of OSA because they are mostly male and have a high incidence of obesity and cardiovascular disease [36,37]. Veterans are prone to smoking and have higher rates of smoking-related disease [38]. US adults have had an estimated 14 million major medical conditions attributable to smoking [39]. Another recent study indicated that smokers were more likely to develop chronic low back pain than nonsmokers [40]. As many as 2/3 of OEF/OIF Veterans complain of insomnia. Older Veterans of prior conflicts report insomnia since their initial service, which suggests a chronic nature to insomnia [41].

There are many modifications patients can make to their lifestyle to help their emotional state and chronic pain, including losing weight, adhering to a CPAP machine, tobacco cessation, and having better sleeping habits. Clinical health psychologists can also provide education and referrals in these areas.

*Weight Loss*. The estimated co-occurrence of pain and obesity in the general population is about 30% according to healthcare claims [42]. The rate of obesity in Veterans is higher, which explains how necessary it is to address obesity when dealing with patients who suffer from pain. Past research findings indicate that there is a linear relationship between weight and musculoskeletal pain, including neck, back, hip, knee, and ankle pain [43]. Obesity is also theorized to affect pain by excess mechanical stresses, which can lead to knee and low back pain [44,45]. Obesity has been correlated with a pro-inflammatory state that can lead to thoracic spine, neck, upper extremity pain, fibromyalgia, migraines, and headaches [46]. Thus, weight loss can reduce chronic pain. For example, one study discovered that more than a 10% loss of body weight in patients diagnosed with obesity resulted in a 50% decrease in knee osteoarthritis [47]. In the converse, chronic pain may lead to obesity because of physical inactivity and the overconsumption of food to provide an anesthetic effect [48,49].

*CPAP Adherence*. The high incidence of obesity in Veterans also underlines the need to address CPAP adherence with patients who suffer from sleep apnea. Untreated OSA can lead to hypertension, cardiovascular disease, mood changes, and memory problems. Using a continuous positive airway pressure (CPAP) device is the most common and efficacious way of treating OSA. Patients tend to become nonadherent to CPAP for many different reasons, including when (1) they have trouble acclimating to the mask’s size or style, which may cause skin irritation or pressure sores; (2) they feel claustrophobic; (3) they have dry mouth or a stuffy nose due to the forced air; (4) they remove the mask during sleep unintentionally; (5) they are irritated by the sound of the CPAP machine; (6) they have difficulty falling asleep; and/or (7) its cleaning and maintenance [50]. All these reasons present different avenues of entry to address this behavior.

*Tobacco Cessation*. Medical experts propose that smoking can interfere with pain management. They report it negatively affects pain in several different ways, including (1) by causing or making painful medical conditions worse; (2) causing the deterioration of spinal discs, which affects chronic neck and back pain; (3) contributing to arthritis and other joint pain; (4) increasing pain sensitivity and affecting the perception of pain; (5) causing increased perception of acute pain; (6) meddling with pain medication; and (7) needing to take larger doses of medications to reduce or manage pain. The US Preventative Services Task Force recommends that providers ask adult patients about tobacco use and provide cessation interventions to users of these products [51]. The treatment for tobacco cessation may include counseling and nicotine replacement therapy, or NRT, such as lozenges, patches, gum, inhalers, and sprays.

*Better Sleep Habits*. There are several likely causes of chronic insomnia, including (1) chronic illness; (2) shift work; (3) poor sleep hygiene; (4) consumption of excess substances (i.e., alcohol or caffeine); (5) being a side effect from medications; and/or (6) a symptom of another psychiatric condition [52]. There are a number of other treatment options (other than medications) that are available to help patients with their disturbed sleep if underlying health issues or environmental factors cannot be recognized or modified. These treatments include behavioral interventions such as biofeedback [53], meditation [54], and cognitive–behavioral therapy (CBT-I). The CBT-I intervention includes relaxation training, cognitive restructuring, stimulus control, sleep restriction, and sleep hygiene.

*Pain Education Programs.* Clinical health psychologists also have a responsibility to provide evaluation, education, and psychotherapy to help reduce the suffering and long-term health care utilization of patients who have chronic pain. During an initial assessment, patients who suffer from chronic pain may be educated about the chronicity of pain and the necessity for self-management. Patients may also be formally referred to a pain education program. Pain education is a central component in quality multidisciplinary care of patients who suffer from chronic pain. Some hospital systems, such as the Department of Veterans Affairs (VA), offer formal pain education programs. For example, the “Pain Education School” program was originally developed at the current pain center to help address the health education needs of Veterans who suffer from chronic pain. Pain Education School consists of a one-hour introduction class during the first week of the month followed by 12-weeks of one-hour classes (total of 13 h). Classes rotate on a fixed scheduled, with the providers presenting change, not the patients, regardless of their entry point. Over the course of the program, 28 interventions are explained (averaging 20–30 min each). Research findings from the first year of the program suggested that participants in the VA program moved forward in their stage of change, improved their experience of pain, and improved their mood [22].

The current quality improvement effort has some limitations. This study used a one group, descriptive analysis rather than any other research design, which would have been inappropriate, misleading, and/or unnecessarily expensive [55]. All the Veterans were referred to the clinical health psychologist by other VA providers, which does not account for a provider effect nor consider whether Veterans had engaged in other health promotion modalities. The needs of all Veterans with chronic, noncancer pain may differ as the current sample was predominately African American, had a large sample of females, and focused more on a middle-aged group, which may be different from the typical Veteran profile. There are some potential barriers to consider when implementing the current model in other clinical settings, such as resource constraints (e.g., costs of having a clinical health psychologist in a pain clinic) and/or challenges in engaging socioeconomically disadvantaged patients (e.g., costs, transportation barriers, and health literacy) that might hinder the translation of this model into practice. Despite these limitations, the current study is the first known investigation that bridges clinical health psychology and pain management by using the biopsychosocial model to address multimorbidity, thereby providing a timely and clinically relevant framework for multidisciplinary care in a VA pain management setting.

## 5. Conclusions

Current statistics related to multimorbidity indicate the prevalence of patients presenting with multiple health conditions has markedly increased in the last few decades. Thus, it is important for providers to know the difference between multimorbidity and comorbidity. Clinical health psychologists are especially qualified to use the biopsychosocial model to address multimorbidity. The biopsychosocial model can be adapted across various clinical settings and can inform healthcare policy, promoting more comprehensive and effective interventions [56]. There are several avenues of entry that clinical health psychologists can navigate to help manage and prevent multimorbidity in their clinics, including assessing and addressing medical conditions, mental health, addiction, and lifestyle habits with their patients. Integrated psychological interventions are crucial in pain management due to the significant impact of psychological factors on the patient’s pain experience and overall well-being. These interventions address risk factors like catastrophizing and pain-related fear, but only when barriers, such as stigma, lack of access, and insufficient training of healthcare professionals, are first addressed. Measurable outcomes of integrated psychological interventions include reduced improved functional capacity and enhanced coping skills [57]. The current article delineates a model of how to address multimorbidity in a sample pain clinic. Pain is a perfect example of an ongoing, persistent condition in which multiple chronic physical and mental conditions are present and for which providers can positively affect a patient’s global health status.
